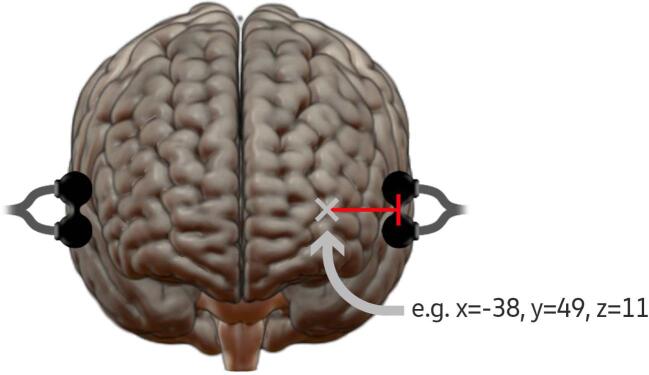# Modulation of dorsolateral prefrontal cortex functional connectivity after intermittent theta-burst stimulation in depression: Combining findings from fNIRS and fMRI

**DOI:** 10.1016/j.nicl.2022.103028

**Published:** 2022-05-02

**Authors:** Wiebke Struckmann, Robert Bodén, Malin Gingnell, David Fällmar, Jonas Persson

**Affiliations:** a Department of Medical Sciences, Psychiatry, Uppsala University, Sweden; b Department of Psychology, Uppsala University, Sweden; c Department of Surgical Sciences, Radiology, Uppsala University, Sweden

**Keywords:** Central executive network, Depressive disorder, Repetitive transcranial magnetic stimulation (rTMS), Resting-state, Salience network

## Abstract

**Background:**

Resting-state functional magnetic resonance imaging (fMRI) can assess modulation of functional connectivity networks following repetitive transcranial magnetic stimulation (rTMS) in the treatment of depression. Functional near-infrared spectroscopy (fNIRS) is well suited for the concurrent application during rTMS treatment sessions to capture immediate blood oxygenation (oxy-Hb) effects, however limited in spatial resolution.

**Objective:**

To understand the network effects behind such a prefrontal fNIRS response during rTMS, and to test whether the fNIRS signal may be predictive of treatment response, we linked data from fNIRS and fMRI within a clinical intervention study.

**Methods:**

42 patients with ongoing depression were recruited and randomized to receive active or sham intermittent theta-burst stimulation (iTBS) over the dorsomedial prefrontal cortex (dmPFC) twice daily for ten days at target intensity. Oxy-Hb was recorded with fNIRS during the first, fifth, and final day of iTBS, with the probe holders located laterally to the TMS coil over regions corresponding to the left and right dorsolateral prefrontal cortex (dlPFC). Resting-state fMRI scanning was performed before and after the whole iTBS treatment course. Functional connectivity analyses were then performed using dlPFC seeds from parcels of a brain atlas showing most overlap with the fNIRS probe locations during treatment.

**Results:**

After active iTBS, left dlPFC-connectivity to the right insula/operculum was reduced compared to sham. The left insula showed a connectivity reduction to the left dlPFC that correlated with an improvement in symptoms. In addition, the posterior parietal cortex showed a connectivity reduction to the left dlPFC that correlated with the fNIRS signal following active iTBS. Finally, the fNIRS oxy-Hb signal from the left dlPFC-seed during the first treatment day was predictive of dlPFC-connectivity change to precentral and temporal cortex regions.

**Conclusion:**

By linking findings from these two different methods, this study suggests that changes within both the salience network and the central executive network affect the fNIRS response to iTBS.

## Highlights

• We recorded oxy-Hb changes during iTBS using fNIRS over the dlPFC.
• This dlPFC region was used as seed in resting-state functional connectivity fMRI.
• Symptom improvement was associated with reduced dlPFC-connectivity to the insula.
• fNIRS oxy-Hb increase was associated with reduced dlPFC-connectivity to the PPC.
• Combining fNIRS and fMRI may facilitate understanding the hemodynamic response to iTBS.

## 1 Introduction

Resting-state functional connectivity (FC) is an MRI-based method that analyzes temporal correlations of spontaneous signals between spatially distinct brain regions. The magnitude of FC within and between brain networks is altered in depression (Li et al., 2018), and baseline FC has been shown to be predictive of treatment response, both for pharmacological treatment as well as repetitive transcranial magnetic stimulation (rTMS) (Dichter et al., 2015). As such, a higher baseline subgenual anterior cingulate cortex (sgACC) connectivity to the dorsolateral prefrontal cortex (dlPFC) and dorsomedial prefrontal cortex (dmPFC) is reported to predict a greater symptom reduction after rTMS (Salomons et al., 2014). A recent meta-analysis confirmed the predictive qualities of the sgACC connectivity for response to standard high-frequency rTMS treatment protocols (Long et al., 2020).

Intermittent theta-burst stimulation (iTBS) is a newer form of rTMS with a much shorter application duration but comparable clinical effectiveness to the standard protocols (Blumberger et al., 2018). In a recent sham-controlled study of iTBS over the dmPFC by our group, we found a symptom related FC increase between the treatment target and the precuneus (Persson et al., 2020). Furthermore, baseline connectivity between this region and both treatment target and sgACC was predictive of treatment response, highlighting the role of the dmPFC as a crucial node between different brain networks affected in depression (Sheline et al., 2010) and that its connectivity strength can be modulated by rTMS.

Functional magnetic resonance imaging (fMRI) is a well-established method for measuring FC by correlating fluctuations in cortical blood oxygenation (oxy-Hb), which is considered a proxy for neural activity due to neurovascular coupling. Since FC can only be measured while lying still in an MRI machine with a strong static magnetic field, other imaging or neurophysiological methods such as functional near-infrared spectroscopy (fNIRS) are better suited for the concurrent application during rTMS treatment sessions to capture immediate effects. fNIRS is an optical imaging method that also measures changes of oxy-Hb, by applying near-infrared light emitter and detector probes on the surface of the head (Scholkmann et al., 2014). This makes it a practical method to study physiological response in real-time during rTMS treatment (Curtin et al., 2019). In another recent study by our group (Struckmann et al., 2020), we used a 2-channel fNIRS device to measure the oxy-Hb response during treatment sessions with iTBS over the dmPFC in a sample of patients with depression. We found that active, but not sham, iTBS modulates the local prefrontal oxy-Hb response over the treatment course, with an increase in oxy-Hb after one and two weeks of iTBS. However, these fNIRS findings are limited in their spatial resolution and coverage, providing no information about potential underlying brain network changes. Combining these fNIRS data with fMRI connectivity analyses would allow for linking concurrent local immediate stimulation effects to more global network effects after a whole treatment course. Furthermore, being able to use the local prefrontal oxygenation signal as biomarker for treatment-related neuronal effects may be a step towards improving the clinical utility of neuroimaging methods with a comparably low cost and simple application, such as fNIRS.

Here, we recorded the fNIRS signal from regions corresponding to the left and right dlPFC, while targeting the dmPFC with the rTMS treatment. This dlPFC region was then used as a basis for defining the seeds for the fMRI resting-state FC analyses. Thus, the aim of this study was to investigate the network effects behind the prefrontal fNIRS response to iTBS. As the fMRI measurements took place at baseline and two weeks after the last iTBS treatment session, we tested for delayed treatment effects and related these to the concurrent effects seen in the fNIRS oxy-Hb response. Furthermore, we explored whether the fNIRS signal assessed early in the iTBS treatment is predictive of fMRI resting-state FC changes. Given the novel approach of linking findings from the two neuroimaging modalities, i.e., fNIRS and fMRI, all analyses were explorative and there were no a-priori hypotheses.

## 2 Materials and methods

### 2.1 Participants

This project shares data with a recent randomized clinical trial comprising patients with depression or schizophrenia (Bodén et al., 2021) and its add-on brain-imaging study (Persson et al., 2020), with all patients being recruited from the psychiatric outpatient clinic at Uppsala University Hospital, Sweden. Out of 101 eligible patients from these two studies, 69 were finally randomized (not meeting the inclusion criteria: n = 5, declined to participate: n = 26, other reasons: n = 1). Out of these 69 patients with depression or schizophrenia, 42 were diagnosed with depression and part of the add-on neuroimaging study, and were thus included in the present study (i.e., the other 27 patients were either diagnosed with schizophrenia or not part of the add-on brain imaging study). All patients included in this study met criteria for an ongoing depressive episode as verified through a Mini International Neuropsychiatric Interview (M.I.N.I.) version 6.0.0 (Sheehan et al., 1998). Inclusion criteria comprised being between 18 and 59 years of age, with unchanged medication one month before treatment start. Medication was kept constant throughout the study. Exclusion criteria comprised epilepsy, intracranial metal implants, active substance use disorder, benzodiazepine use, and pregnancy. Written informed consent was obtained by all patients prior to study participation. The work described has been carried out in accordance with the Declaration of Helsinki and the study was approved by the Ethical Review Board in Uppsala.

### 2.2 Procedures

**Supplementary figure 1:**
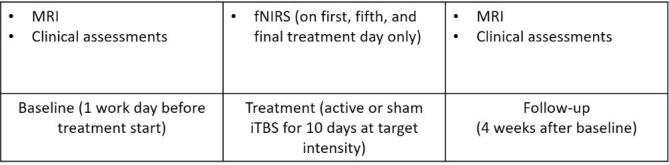


The patients were randomized to receive active or sham treatment in a blind treatment phase, with the iTBS protocol described below (*2.3 Intermittent theta-burst stimulation*). FNIRS was recorded during the first, fifth, and final day of iTBS treatment (*see 2.4 fNIRS acquisition*). Clinical assessments of depression symptoms, such as the Clinical Assessment Interview for Negative Symptoms (CAINS) (Kring et al., 2013) or the Montgomery Åsberg Depression Rating Scale (MADRS) (Svanborg & Åsberg, 1994), and functional magnetic resonance imaging (fMRI) were conducted one work day before treatment start (baseline), and once again four weeks later (follow-up). Thus, the follow-up did not take place immediately after the last iTBS session, but rather two weeks later to assess potential delayed effects. An overview of the study design is depicted in Supplementary Fig. 1.

### 2.3 Intermittent theta-burst stimulation

The iTBS protocol is described in more detail in (Bodén et al., 2021). Treatment was delivered using the Magpro X100 with Magoption and the Cool D-B80 A/P coil from MagVenture, Farum, Denmark. This device comprises two sides with identically looking coils, with the sham coil being internally shielded. To ensure blindness of the operator, a randomization code entered into the TMS apparatus decided which side of the coil to be angled towards the patient. Using each subjects’ baseline MRI image and the Localite TMS Navigator (Localite, Bonn, Germany), neuronavigated iTBS was delivered over the dmPFC, with the dorsal anterior cingulate cortex (Montreal Neurological Institution (MNI) coordinates x,y,z = [0,30,30] (Mir-Moghtadaei et al., 2016)) as the main target area. The treatment was given for ten days of target intensity, defined as 90% of the patient’s individual resting foot motor threshold, with two sessions per day separated by a fifteen-minute intersession interval (Tse et al., 2018). Each session comprised 40 trains of stimulation, with each train consisting of two seconds stimulation, and eight seconds off. The stimulation comprised ten bursts at 5 Hz, and three biphasic pulses at 50 Hz per burst, thus delivering 1200 pulses per session. For all patients, transcutaneous electrical nerve stimulation (TENS) electrodes were applied directly under the TMS coil. These were not activated in the active treatment group. In the sham group, a mild TENS was delivered synchronously to the magnetic pulses and in proportion to iTBS stimulation intensity, with a maximum current of 4 mA, to mimic the sensation of the active treatment.

### 2.4 fNIRS acquisition

As described in detail in (Struckmann et al., 2020), blood oxygenation was measured with fNIRS on the first, fifth, and final day of iTBS treatment with a two-channel CW-NIRS device (two LEDs, λ_1|2|3_ = 735|810|835 nm with average power less than 2mW, two photodiode detectors, emitter-detector distance: 3.5 cm) (NIRO 200X, Hamamatsu, Japan). The signal was recorded throughout the iTBS sessions at a sampling frequency of 5 Hz. The fNIRS probe holders were applied lateral to the TMS coil, on the left and right forehead. The optodes’ emitter and detector positions were recorded using neuronavigation (Localite, Bonn, Germany). The fNIRS signal acquisition started once the patient was seated comfortably in the treatment chair and an initial resting-state measurement was conducted for five minutes to allow for signal equilibrium. Signal acquisition was continuous over both treatment sessions and the fifteen-minute intersession interval, with patients being instructed to sit calmly and rest. During the stimulation, the TMS device sent trigger signals for the onset of each TMS burst to the NIRO device.

### 2.5 fNIRS data analyses

Data analyses has been described in detail in (Struckmann et al., 2020). Preprocessing and data analyses were performed in MATLAB12. For preprocessing, the fNIRS data were band-stop filtered (0.12–0.35 Hz and 0.70–1.5 Hz for removal of noise stemming from respiration and heartbeats, respectively). For subsequent data analysis, individual data segments were taken from the eight seconds off within each iTBS train (*2.3 Intermittent theta-burst stimulation*), resulting in 80 segments for each patient on each treatment day. The individual means for each iTBS train were averaged and the mean peak of the active group plus/minus 1 s was used as interval in the analysis. The individual means for each treatment day (first, fifth, and final) were subsequently entered in a linear mixed effect (LME) model for each fNIRS channel (left and right) with the factor group (active vs. sham) as fixed effect, testing for different trajectories between the groups indicated by a day × group interaction effect. Additionally, pairwise comparisons between the groups at each treatment day were calculated. To test whether changes in the oxy-Hb response were associated with symptom change, Pearson correlation analyses were conducted for the full patient sample. To test whether the oxy-Hb response from the first treatment day was predictive of symptom change, Pearson correlation analyses were conducted within the active iTBS group. The threshold for significance was set at p =.05.

### 2.6 MRI acquisition

MRI was performed using a 3 T scanner (Philips Achieva, Philips Medical Systems, Best, The Netherlands) with a 32-channel head coil. Scanning took place at Uppsala University Hospital, Sweden. An anatomical T1-weighted image was acquired using a 3D multi-shot spin echo sequence (TR/TE 8.2/3.8 ms, flip angle = 8°, field of view 256x256 mm^2^ with voxel sixe 1x1x1mm^3^). For FC analysis, a BOLD T2*-weighted image sequence was acquired using a single-shot EPI sequence (TR/TE 2000/30 ms, flip angle = 90°, field of view 256 × 256 mm^2^ with voxel size 3x3x3 mm^3^, interleaved acquisition). Throughout this seven-minute scanning interval, patients were instructed to direct their gaze towards a white fixation cross on a black screen, using fMRI compatible goggles (NordicNeuroLab, Bergen, Norway), and rest.

### 2.7 MRI data analyses and statistical procedures

The fMRI data were preprocessed in the CONN toolbox, using its default preprocessing pipeline for volume-based analyses in MNI space, including realignment, slice-timing correction, normalization, ART-based outlier detection, smoothing of the functional data, and segmentation and normalization of the anatomical data. To remove motion related and physiological artefacts, data was then denoised by applying a linear regression model with confounders (i.e., components of white matter, CSF, realignment, and scrubbing) and the main task effects, and a temporal band-pass filter of 0.008–0.09 Hz. Patients were excluded if less than 125 frames remained after scrubbing in any of the two scanning sessions (Power et al., 2012).

**Fig. 1:**
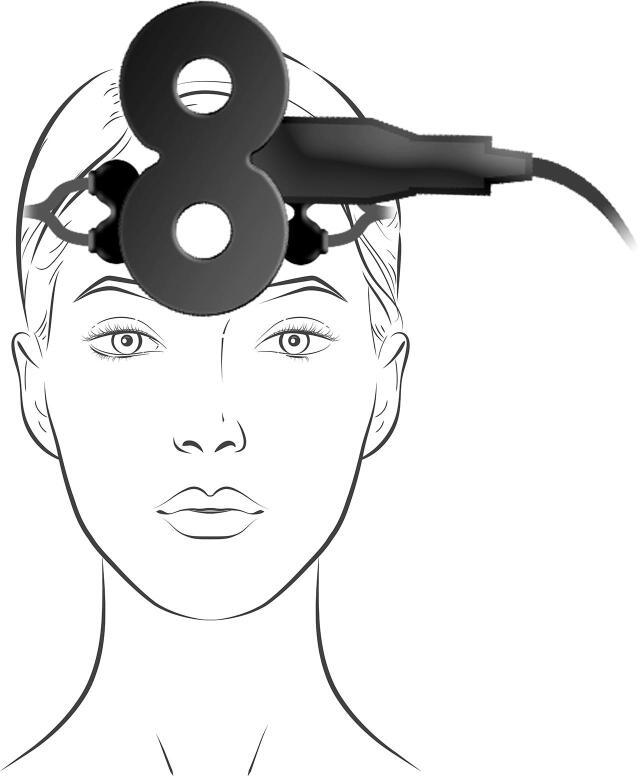

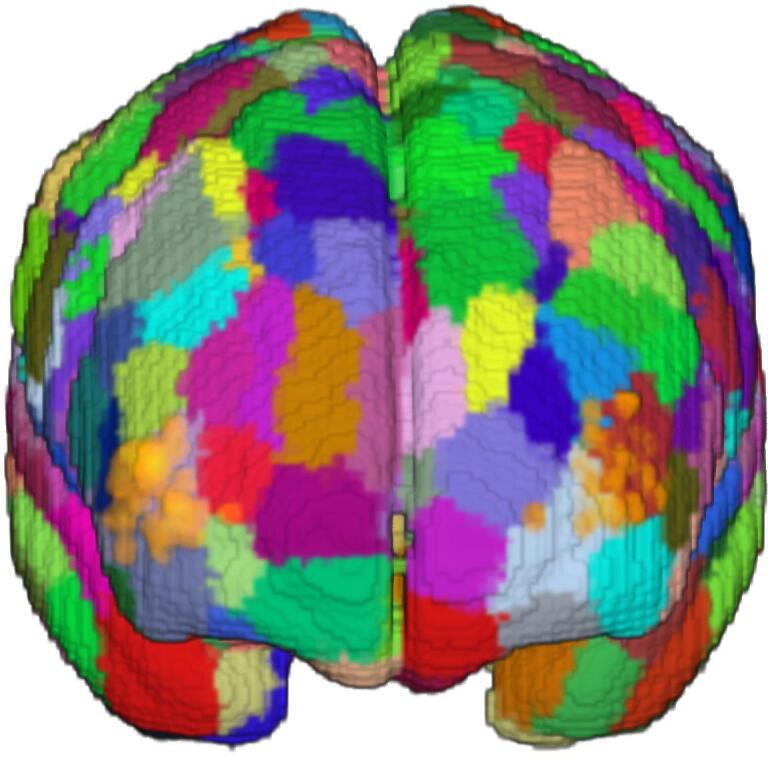
*1A:* Placement of fNIRS optodes on the forehead, with the light emitter above the detector, aligned with the coil. *1B:* fNIRS optode center locations (orange dots) onto the 400 parcellation atlas by Schaefer et al. (2018). The parcel with the highest optode center load per hemisphere was used as region of interest in the functional connectivity analyses, corresponding to regions within the left and right dorsolateral prefrontal cortex. (For interpretation of the references to colour in this figure legend, the reader is referred to the web version of this article.)

For first-level analyses, seed-to-voxel functional connectivity analysis was performed for each patient’s baseline and follow-up scan in CONN, and the resulting connectivity correlation values were transformed using Fisher’s r-to-z transformation. To calculate difference correlation maps reflecting changes in connectivity after the iTBS treatment, the baseline correlation map was subtracted from the follow-up correlation map for each patient. The seed regions of interest (ROIs) were chosen according to the fNIRS optodes’ emitter and detector placement: To project the optode positions onto the brain surface, individual T1-weighted images were first segmented using SPM12. The center coordinate of the emitter-detector distance was then calculated, and the closest voxel within the grey matter segmentation was identified. The respective coordinates were then transformed into MNI space, using the deformations obtained from the segmentation step. These optode center coordinates were then classified according to Schaefeŕs 400 parcellation atlas (Schaefer et al., 2018) (Fig. 1), an atlas derived from resting-state functional connectivity data. On the left hemisphere, 52% of the optode centers were overlapping with parcel 137 (center of mass coordinates X  = -38, Y = 49, Z = 11) and 17% with parcel 135 (center of mass coordinates X  = -42, Y = 48, Z = -6). The remaining parcels had an overlap with less than 10 % of the optode centers. On the right hemisphere, 77% of the optode centers were overlapping with parcel 345 (center of mass coordinates X  = 42, Y = 46, Z = 14), and 51 % with parcel 343 (center of mass coordinates X = 27, Y = 59, Z = 3). The parcels correspond to the dlPFC in the atlases’ control network (Schaefer et al., 2018). The dlPFC parcel containing the largest optode center load per hemisphere was used as seed ROIs. Combining the two right dlPFC-parcel seeds did not change the results in a statistically meaningful way.

Second-level analyses were performed in SPM12. To assess baseline dlPFC connectivity in the full patient sample, a one-sample t-test with the baseline correlation maps was conducted. To test whether baseline dlPFC connectivity was predictive of symptom change following active treatment, a multiple regression on the baseline connectivity maps with symptom change as covariate was conducted within the active group. To test for group differences (i.e., active vs. sham) in functional connectivity changes after treatment, a two-sample *t*-test with the difference maps was conducted. This was followed up with testing for symptom-related modulation of seed connectivity between groups, by adding CAINS change (follow-up minus baseline) as a factor. Similarly, we tested whether change in the fNIRS response over the treatment course was related to modulation of dlPFC connectivity, following active compared to sham iTBS. Here, a two-sample t-test, i.e., active vs. sham, was conducted on the FC difference maps with the fNIRS mean signal change (final day minus first day) as an additional factor. This was followed up with within-group analyses, testing for fNIRS signal-related modulation of seed connectivity in the active and the sham group separately. Finally, we tested whether the initial fNIRS response (i.e., from the first treatment day) is predictive of dlPFC connectivity modulation following active iTBS. Here, a multiple regression was conducted with the FC difference maps of the patients in the active group and their fNIRS mean signal from the first treatment day as covariate. For all connectivity analyses containing fNIRS data, only ipsilateral fNIRS channel-seed combinations were used, i.e., the left fNIRS channel with the left dlPFC seed, and the right fNIRS channel with the right dlPFC seed. Voxels surviving a cluster-level corrected threshold of p <.05 were considered significant, using an initial cluster-forming threshold of p <.001 to correct for multiple comparisons.

For analysis of the clinical data, CAINS scores from the different time points (baseline vs. follow-up) were entered in an LME model with the factor group (active vs. sham) as fixed effect, testing for different trajectories between the groups indicated by a time × group interaction effect.

## 3 Results

Forty-two patients participated in the study and were allocated to active (n = 21) or sham (n = 21) iTBS treatment. Eight patients were excluded from the analysis due to a missing follow-up session (n = 2) or the fMRI data being corrupted by too many motion artefacts (n = 6). This resulted in 17 patients in the active iTBS group, and 17 patients in the sham iTBS group included in the analysis. The groups did not differ in their sociodemographic and clinical characteristics (Table 1). The change in CAINS score (baseline – follow-up) was 6.5 ± 8.5 points for the active treatment, and 6.9 ± 10.8 points for the sham treatment. An LME model showed no significant time (baseline vs. follow-up) × group (active vs. sham) interaction (t = -0.12, p =.902).

**Table 1:** Sociodemographic and clinical characteristics at baseline for patients allocated to active or sham intermittent theta-burst stimulation. *CAINS*: Clinical Assessment Interview for Negative Symptoms; *MADRS-S*: Montgomery Åsberg Depression Rating Scale; *BPRS*: Brief Psychiatric Rating Scale; *MSM*: Maudsley Staging Method for treatment resistant depression; *EQ-5D VAS*: self-rated health from EQ-5D, Visual Analogue Scale; *ADHD*: attention deficit hyperactivity disorder; *ADD*: attention deficit disorder.

	Active (n = 17)	Sham (n = 17)	Test for difference
Years of age, mean (sd)	30.9 (10.3)	27.1 (7.2)	t(32) = 1.20, p =.241
Sex, female:male	9:8	9:8	χ^2^ = 0.00, p = 1.000
CAINS, mean (sd)	27.2 (7.2)	28.9 (7.5)	t(32) = -0.66, p =.517
MADRS-S, mean(sd)	29.0 (6.7)	29.1 (7.2)	t(32) = -0.05, p =.962
BPRS, mean (sd)	49.9 (8.5)	50.9 (5.4)	t(32) = -0.40, p =.693
MSM, mean (sd)	9.5 (1.56)	10.2 (1.7)	t(32) = -1.32, p =.196
EQ-5D VAS, mean (sd)	31.9 (15.4)	34.9 (13.5)	t(32) = -0.59, p =.561
Education, n			χ^2^ = 1.54, p =.462
9th year completed	2	4	
12th year completed	8	9	
Higher education	7	4	
Primary diagnosis, n			χ^2^ = 0.39, p =.825
Depressive episode	10	9	
Recurrent depression	6	6	
Bipolar disorder	1	2	
Secondary diagnoses, n			
Anxiety disorders	7	7	χ^2^ = 0.00, p = 1.000
ADHD/ADD	3	6	p =.438
Autism spectrum disorders	1	0	p = 1.000
Personality disorders	0	2	p =.4851
Medication, n			χ^2^ = 0.73, p =.866
Antidepressants	15	14	
Mood stabilizers	6	4	
Antipsychotics	3	4	
None	2	1	

### 3.1 Baseline functional connectivity

**Fig. 2:**
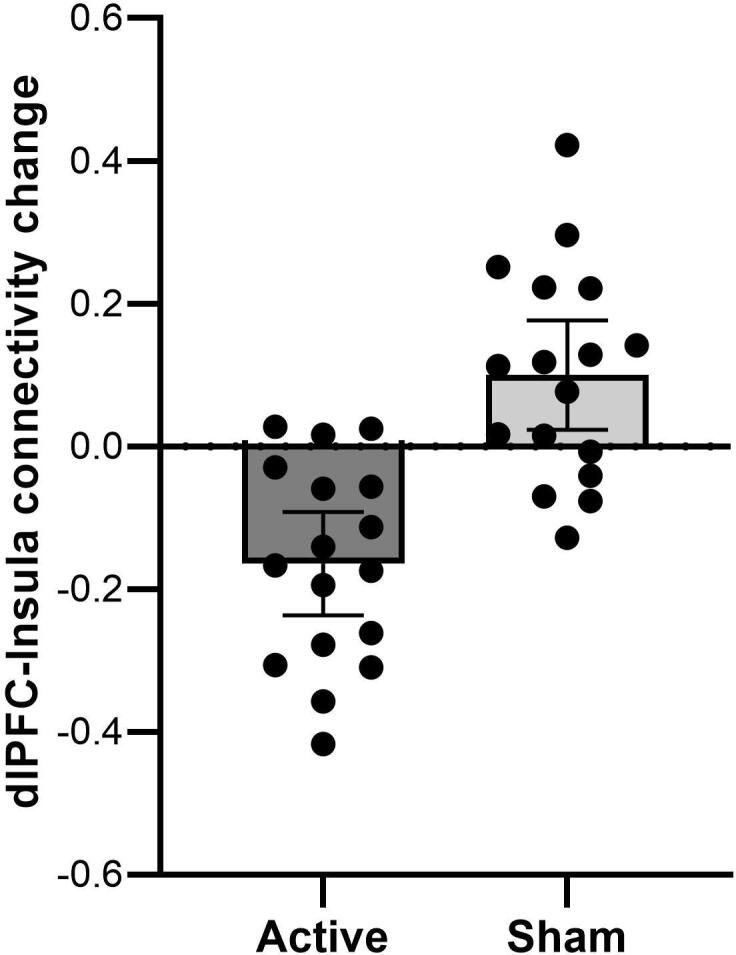
Group means and individual data points depicting left dlPFC-connectivity changes to a cluster spanning the right insula and operculum (peak at X = 46, Y = 4, Z = 2, t = 4.44, p =.001, cluster size = 416) after active and sham iTBS. Note that a positive value indicates an increase in connectivity, and a negative value a decrease in connectivity following iTBS treatment. Error bars mark the 95 % confidence interval.

The left and right dlPFC seeds showed similar connectivity patterns at baseline (Fig. 2). The seeds showed a positive relationship to a large cluster spanning the left and right dlPFC, operculum, and insula, the posterior parietal cortex (PPC), the caudate nucleus, and a cluster spanning the middle and inferior temporal gyrus. In contrast, both seeds showed a negative relationship to the medial PFC, precentral gyrus, hippocampus, amygdala, and middle temporal gyrus. Table 2 lists all clusters with their respective peak coordinates and statistical values. Prediction analyses within the active group showed no relationship between dlPFC connectivity at baseline and symptom change following treatment.

**Table 2:** Baseline resting-state functional connectivity of the dorsolateral prefrontal cortex seeds. *FC:* functional connectivity; *dlPFC:* dorsolateral prefrontal cortex; *PPC:* posterior parietal cortex; *MTG:* middle temporal gyrus; *PCC:* posterior cingulate cortex; *ITG:* inferior temporal gyrus; *SFG:* superior frontal gyrus; *MCG:* middle cingulate cortex; *mPFC:* middle prefrontal cortex; *MFG:* middle frontal gyrus.

	Region	Peak coordinates [x,y,z]	peak-level *T*	cluster-level *p*_FWE-corr_	Cluster size
Left dlPFC parcel					
Positive FC	Cluster incl. dlPFC, operculum, insula	−42,48,8	39.37	0.000	239 210
	MCG	0,2,30	7.92	0.000	1 445
	Caudate nucleus	12,2,14	7.17	0.000	942
	PPC, l	−52,-42,46	14.53	0.000	102 400
	Cluster incl. MTG, ITG, l	−56,-58,-6	11.40	0.000	5 383
	ITG, r	58,-54,12	8.13	0.000	1 865
	Cerebellum, r	30,-64,-34	6.09	0.000	1 247
Negative FC	mPFC	−2,60,18	10.05	0.000	3 561
	Cluster incl. temporal pole, hippocampus	44,4,-34	9.28	0.000	118 910
	MTG, l	−56,-10,-16	8.63	0.000	2 365
	Precentral g, r	20,-34,80	6.32	0.000	557
	Cerebellum	−2,-56,46	5.96	0.003	343

Right dlPFC parcel					
Positive FC	Cluster incl. dlPFC, operculum, insula	44,46,14	35.74	0.000	542 410
	MFG, l	−26,4,62	6.10	0.000	575
	Caudate, l	−10,4,12	6.63	0.000	461
	ITG, r	54,-44,-20	9.90	0.000	2 121
Negative FC	mPFC	−2,64,10	12.51	0.000	219 280
	MTG, r	62,-2,–22	8.76	0.000	1 973
	Precentral g, r	32,-24,74	5.72	0.021	232
	Hippocampus, l	−34,-18,-16	10.22		
	Cerebellum	4,-54,-48	9.80	0.000	596
	Cerebellum	24,-78,-36	7.26	0.003	338

### 3.2 fNIRS changes

An LME model for the left fNIRS channel showed a day × group interaction (t = 2.13, p =.035). Pairwise comparisons between the groups showed a difference on the fifth (t = 4.37, p <.001) and final (t = 3.04, p =.005) day, with higher oxy-Hb values in the active iTBS group compared to sham. For the right fNIRS channel, an LME model did not show a day × group interaction (t = 0.75, p =.455). Pearson correlation analyses showed no relationship between change in oxy-Hb and change in clinical symptoms (left fNIRS channel: r =.-0.06, p =.731. right fNIRS channel: r = 0.05, p =.773). For prediction analyses within the active group, Pearson correlation analyses showed no relationship between the oxy-Hb response from the first treatment day and change in clinical symptoms (left fNIRS channel: r = -0.05, p =.847, right fNIRS channel: r = -0.07, p =.806).

### 3.3 Functional connectivity changes after iTBS

**Fig. 3:**
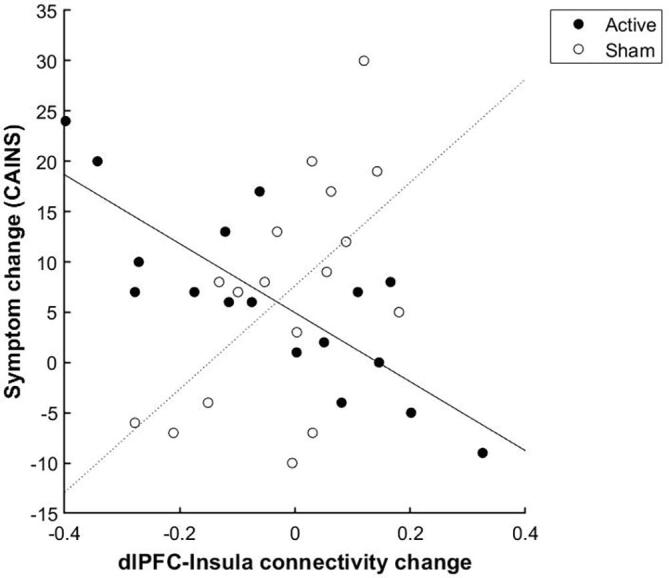
Active compared to sham iTBS was followed by a stronger negative relationship between left dlPFC connectivity change and symptom change in the left insula (X = -36, Y = -10, Z = 24, t = 5.46, p =.022, cluster size = 226). Note that a positive symptom change value indicates a symptom reduction.

After active compared to sham iTBS, there was a greater reduction in left dlPFC connectivity to a cluster spanning the right insula and operculum (X = 46, Y = 4, Z = 2, t = 4.44, p =.001, cluster size = 416) (Fig. 3). Adding symptom change as a regressor, there was a stronger negative relationship between left dlPFC connectivity change and symptom change in the left insula (X = -36, Y = -10, Z = 24, t = 5.46, p =.022, cluster size = 226) after active compared to sham treatment, indicating greater symptom improvement with greater reduction in connectivity between these regions (Figure 4).

### 3.4 fNIRS signal-related connectivity changes

There was a negative relationship in the active group between left dlPFC connectivity and fNIRS signal change in the posterior parietal cortex (PPC) (specifically, the superior parietal lobule with peak coordinates X = 34, Y = -48, Z = 56, t = 6.68, p =.027, cluster size = 164), indicating a greater fNIRS signal increase during the treatment course was followed by a greater reduction in connectivity between these regions. No significant correlation was found in the sham group for either left or right dlPFC. However, in a direct comparison of the active and sham groups, there was no significant difference in the relationship between dlPFC connectivity change and fNIRS signal change.

### 3.5 fNIRS signal predictive of connectivity change after active iTBS

There was a positive relationship between left-channel fNIRS during the first iTBS treatment day and left dlPFC connectivity change within the left and right precentral gyrus (left: X = -40, Y = -14, Z = 52, t = 6.21, p =.004, cluster size = 236, right: X = 44, Y = -12, Z = 60, t = 7.42, p =.002, cluster size = 267), the left superior temporal gyrus (X = -64, Y = -16, Z = -4, t = 12.37, p =.002, cluster size = 279), and the inferior temporal gyrus (X = -56, Y = -34, Z = -30, t = 6.36, p =.018, cluster size = 178).

## 4 Discussion

In this randomized sham-controlled study, we recorded blood oxygenation using fNIRS from a region within left and right dlPFC during dmPFC-iTBS treatment. Using these dlPFC regions as seed in resting-state fMRI connectivity analyses, we show that after active compared to sham iTBS, symptom improvement was associated with reduced connectivity to the insula, while fNIRS oxy-Hb increase during active treatment was associated with reduced connectivity to the PPC. fNIRS oxy-Hb recorded during the first treatment day was predictive of dlPFC-connectivity change to precentral and temporal cortex regions.

While symptom improvement did not differ between the groups, there were greater FC modulations after active compared to sham treatment. Our findings of reduced frontoinsular connectivity after active iTBS are in line with reports of single-session iTBS over the left dlPFC dampening connectivity to the insula in healthy controls (Iwabuchi et al., 2017), and we expand this finding to a new treatment target within an antidepressive treatment setting. A symptom-related frontoinsular FC modulation is also in line with Salomons et al. (2014) who showed that greater reduced connectivity between the dmPFC and bilateral insula was associated with higher iTBS treatment response (Salomons et al., 2014). Our results extend this observation of anticorrelation of prefrontal-insular connectivity and clinical improvement to a sham-controlled setting. Being a key node in the salience network (SN) (Seeley et al., 2007), but also highly interconnected with other brain networks, connectivity to the insula is often altered in depression (Manoliu et al., 2014). As such, high functional connectivity within the SN (Ge et al., 2017), and high network segregation of the SN (Fan et al., 2019) have been reported to be predictive of rTMS treatment response. These findings suggest an important role of the SN in mediating symptom reduction in depression, which is a pattern we also observed within the present study, and may point to a treatment-specific network effect.

Furthermore, our results might link SN changes to the dlPFC. The dlPFC, together with the PPC, are primary nodes in the central executive network (CEN) (Seeley et al., 2007). The CEN is predominantly characterized by hypoconnectivity in depression (Kaiser et al., 2015). Regarding rTMS-induced FC changes, the CEN is not as well described as the SN and DMN; however, there are reports of unchanged CEN connectivity after a full rTMS treatment course (Liston et al., 2014). However, Zheng et al. (2020) report increased CEN connectivity density, a graph-based indicator of global FC, following rTMS treatment (Zheng et al., 2020), suggesting that the CEN may be modulated by rTMS in depression.

The observed changes in FC following iTBS treatment point to different brain networks known to be altered in depression, namely the SN (Manoliu et al., 2014) and the CEN (Kaiser et al., 2015): acting through the insula, the SN might be involved in negative symptom reduction, whereas the oxy-Hb modulation captured with fNIRS potentially reflects changes within the CEN. Findings from a recent multi-dataset study point to nodes of both networks being a part of a global depressive circuit that is responsive to rTMS treatment (Siddiqi et al., 2021). It is yet unclear however, to which extent the connectivity changes within the SN and the CEN observed in the present study reflect distinct processes or if both contribute to a global antidepressive response. Here we observe reduced connectivity between nodes of CEN and SN following treatment. It is possible that this reflects an increased segregation of these two networks necessary for optimal functioning, although we did not measure this directly.

To the best of our knowledge, this study is the first to explore whether oxy-Hb fNIRS signal recorded during the first active iTBS treatment day is predictive of connectivity changes of the cortical fNIRS probe location. We found prediction effects for dlPFC-connectivity change to precentral and superior and inferior temporal cortex regions. These regions are not included in either SN, CEN, or DMN, making interpretation of the results somewhat difficult. Hitherto, most fNIRS research in clinical settings measures task-related oxy-Hb concentration changes to differentiate signal patterns between depressed and healthy individuals (Ho et al., 2020), and only few studies have conducted resting-state FC analyses with fNIRS. Those studies report a global disrupted FC in depression (Zhu et al., 2017). For altered frontoparietal FC with fNIRS, there are inconsistent findings on the direction of FC change and whether this is associated with symptom improvement (Rosenbaum et al., 2016, Sakakibara et al., 2021). Thus, while interpreting the direction of the present fNIRS prediction effects is not yet possible, combining findings from fNIRS and fMRI is a promising approach for obtaining a more complete picture of the hemodynamic response. As such, a recent study showed that the fNIRS signal recorded during simple motor and language tasks corresponds fairly well to the fMRI signal of the same tasks, recorded at the same day (Wagner et al., 2021), highlighting the potential of fNIRS as a useful measure of concurrent brain activity during rTMS.

When interpreting the findings from the present study, precaution is needed as the two methods for measuring blood oxygenation used here are based on different modalities. Also, we recorded the fNIRS signal simultaneously to the iTBS sessions, whereas the fMRI connectivity data stem from resting-state measurements before and after the treatment course. However, this study did not aim to treat these methods as interchangeable but by combining them, to describe the network effects of the fNIRS response to iTBS. Future research actually combining the two imaging methods during rTMS is warranted. Further limitations of the present study is that the sample size was moderate, hampering the generalizability of the findings.

In conclusion, this study is the first to link fNIRS findings to resting-state fMRI connectivity analyses in the context of antidepressive iTBS treatment. The results suggest an involvement of the SN and the CEN behind the observed oxy-Hb increase following active iTBS.

## 5 Data and code availability statement

Data are available from the corresponding author (WS) upon reasonable request.

### CRediT authorship contribution statement

**Wiebke Struckmann:** Data collection and analysis, Writing – original draft, Writing – review & editing. **Robert Bodén:** Conceptualization, Funding acquisition, Project administration, Writing – review & editing. **Malin Gingnell:** Conceptualization, Writing – review & editing. **David Fällmar:** Methodology, Writing – review & editing. **Jonas Persson:** Conceptualization, Funding acquisition, Writing – review & editing.

## Declaration of Competing Interest

The authors declare that they have no known competing financial interests or personal relationships that could have appeared to influence the work reported in this paper.

## Figures and Tables

**Supplementary figure 2:**